# Has “respiratory coaching” before deep inspiration an impact on the incidence of transient contrast interruption during pulmonary CT angiography? 

**DOI:** 10.1007/s13244-012-0182-z

**Published:** 2012-07-07

**Authors:** Juan M. Bernabé-García, Cristina García-Espasa, Juan Arenas-Jiménez, José Sánchez-Payá, Javier de la Hoz-Rosa, Joan O. Carreres-Polo

**Affiliations:** 1 Department of Radiology, General Universitary Hospital of Alicante, Avda. Pintor Baeza 12, 03010 Alicante, Spain; 2 Department of Public Health, General Universitary Hospital of Alicante, Avda. Pintor Baeza 12, 03010 Alicante, Spain

**Keywords:** Tomography, Spiral computed, Angiography, Artifacts, Pulmonary embolism, Randomised study

## Abstract

**Purpose:**

To evaluate if respiratory coaching performed prior to CT pulmonary angiography (CTPA) image acquisition has an impact on the occurrence of transient interruption of contrast (TIC) phenomenon.

**Materials and methods:**

Two hundred and thirty-one consecutive patients with suspected pulmonary embolism (PE) were referred for CTPA. They were randomised into two groups, with or without respiratory coaching (groups A and B, respectively). Those patients who were deemed not able to be coached were not randomised and were assigned to a third group (C). Two radiologists evaluated the degree of enhancement of the pulmonary arteries and the presence and grade of TIC. The χ^2^ test was used to compare differences among groups in occurrence and grade of this phenomenon.

**Results:**

There were no significant differences in the presence of any grade of TIC among the three groups, with 30 positive cases (32%) in group A, 33 (35%) in group B, and 12 (27%) in group C (*P* = 0.61). When TIC was graded and divided into significant or not, the different groups also did not differ significantly.

**Conclusion:**

Performing respiratory coaching before CTPA had no statistically significant effect on the incidence and severity of TIC in this prospective randomised study.

***Main Messages*:**

• *Significant transient interruption of contrast appears in 12% of pulmonary CT angiograms.*

• *Severe transient interruption of contrast leading to nondiagnostic tests appears in 2% of studies.*

• *In our study respiratory coaching has no impact on the incidence of transient interruption of contrast.*

## Introduction

Thin-slice multidetector computed tomographic pulmonary angiography (CTPA) is a technique with well-known sensitivity and specificity (over 90%) for detection of pulmonary embolism (PE) and has become the test of choice for diagnosing acute PE in most institutions [1–3]. However, numerous pitfalls and artefacts have been described that can reduce the accuracy of the CTPA [4]. Transient interruption of contrast (TIC) is a physiological artefact that was first described by Gosselin et al. [5]. This flow artefact consists of a segment of the pulmonary artery that demonstrates relatively poor contrast enhancement in between areas of increased attenuation both proximal and distal to it [6]. It is believed that this vascular phenomenon occurs when the patient performs a deep inspiration just before the scan starts, resulting either in increased venous return of unopacified blood from the inferior vena cava (IVC) or reduced delivery of contrast medium via the superior vena cava [7]. Unopacified blood entering the right atrium dilutes the contrast column coming from the superior vena cava (SVC) [8]. Thus, a transient decrease in attenuation of pulmonary arteries can be seen. In some patients, the presence of a patent foramen ovale (PFO) can play a role by causing a transient intracardiac right-to-left shunt with deep inspiration [9].

Recently, Mortimer et al. [7] demonstrated that expiratory scans have a significantly lower incidence of TIC compared with inspiratory studies. Since this phenomenon seems to be related to respiratory dynamics, some authors have suggested that certain inspiratory maneuvers could potentially decrease the frequency of TIC, but there has been no agreement on a specific protocol, and the effect of performing several respiratory cycles before scan has not been evaluated.

Since there are no publications that address and compare these respiratory protocols, we conducted a prospective randomised study to evaluate if performing respiratory coaching before scanning has a significant impact on the frequency of TIC.

## Materials and methods

### Patient groups and randomisation

Ethics committee approval and signed informed consent were obtained. Between January 2007 and November 2007, 233 consecutive patients referred to our Department with clinical suspicion of PE were enrolled in this prospective study. Before undergoing CTPA, patients were evaluated for their capability to follow the respiratory orders properly. Those who were deemed capable of cooperating were randomised into two groups. In group A, the patients were instructed to take several deep inspirations and expirations just before and during contrast infusion, followed by an inspiration just at the beginning of the scan. The ones in group B were asked to make an inspiratory effort at the beginning of the scan, without previous coaching. Finally, those patients who were considered not able to cooperate formed a third group, group C, and the scan was performed without any respiratory instructions or with the simple instruction that the patient should take a “shallow breath.” From the initial number of patients, 45 were not randomised as they were considered noncooperative and were thus included in group C. Two randomised patients were excluded from the final selection, one of them due to contrast extravasation during CTPA and the other because missing images precluded the evaluation. Both patients belonged to group A.

Of the 231 patients who entered the study, age, sex, weight, and height were registered. Characteristics of patient group A were as follows: 92 patients, 46 men, 46 women; mean age, 60 ± 19 years (standard deviation); range, 19–91 years. There were 94 patients in group B, 53 men, 41 women; mean age, 66 ± 17 years; range, 18–92 years. Characteristics of patient group C were as follows: 45 patients, 20 men, 25 women; mean age, 75 ± 12 years; range, 30–93 years.

### CT pulmonary angiography

All scans were obtained using a MDCT scanner (Siemens Somatom Sensation 10; Siemens, Erlangen, Germany) with 10-detector array. CTPA parameters were as follows: 0.75 mm collimation, 7.5 mm table movement per gantry rotation, 0.5 s per rotation, 120 reference mAs, and 100 kVp. Patients were examined in the supine position with both arms extended above the head. A frontal scout view was acquired at 80 kVp and 50 mA. Scan was obtained in the craniocaudal direction during a single inspiratory breath hold in groups A and B. Patients in group C, considered not able to cooperate, were requested to perform shallow breathing. Scan was performed from above the aortic arch to the level of the right diaphragm.

For all examinations, a typical 100 mL bolus of iodinated contrast material (Iopromide, 370 mg iodine/mL, Ultravist; Bayer Schering, Berlin, Germany) was injected into a peripheral vein in the arm at a flow rate of 3 mL/s. Injections were performed automatically by using a commercially available power injector (Stellant, Medrad, Pittsburgh, PA).

Individual contrast timing was achieved by using bolus tracking (Care Bolus; Siemens, Erlangen, Germany) in the main pulmonary artery with threshold set at 100 Hounsfield units (HU) followed by an 8 s delay for beginning the scan.

Regarding postprocessing, thin-section reconstruction was performed with a section thickness of 1 mm, an increment of 0.7 mm, and a smooth reconstruction kernel (B30f). Final image analysis was blindly assessed by two radiologists at a workstation (EasyVision; Philips Medical Systems, Best, the Netherlands) with an optimised CT angiography window normally adjusted for each patient and dedicated multiplanar reformations. All studies were reviewed by one of the authors with 13 years of experience in chest CT and by one of two other authors with 3 years of experience.

### Assessment of image parameters

#### Vascular attenuation measurements

We evaluated the vascular attenuation in HU (mean value and standard deviation) by placing a circular region of interest (ROI) over the main pulmonary artery, right pulmonary artery, and left pulmonary artery in 3-mm-thickness axial images. Aortic enhancement was also assessed at the level where it appeared higher in density. The two regions of interest used for these measurements were chosen to be at least 1.5 cm^2^.

Discrepancies in measurements were resolved as follows: for figures with less than 10 HU difference, the mean between both measurements was used for analysis; in the remaining cases, images were reviewed, and a joined measurement was obtained to reach a consensus.

#### TIC diagnosis and grading

**Fig. 1:**
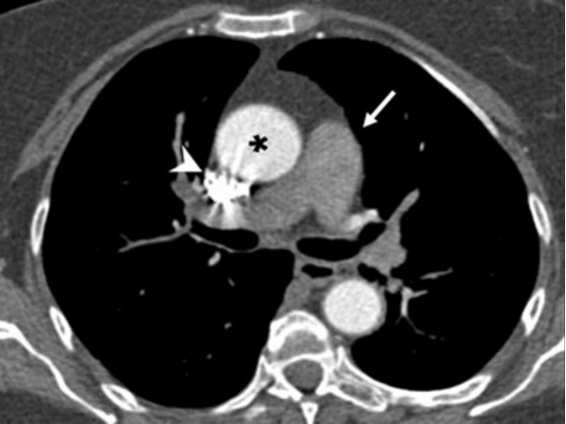
Transverse CT pulmonary angiogram at the level of the right pulmonary artery showing grade 1 transient interruption of contrast (TIC) in an 81-year-old woman. Pulmonary arteries (*arrow*) measure 72 HU, while the aorta (*asterisk*) is properly enhanced and contrast is still present in superior vena cava (*arrowhead*)

Presence or absence of TIC was first subjectively evaluated. After that, HU values were measured in all patients as described before. We considered TIC to be present when a segment of pulmonary arteries showed lower densities (a difference of at least 10 HU) than other portions of the pulmonary arteries (both proximally and distally) and/or the aorta. In addition, a subjective grading of this TIC was made using a five-point scale (Table 1) with score 1 corresponding to the most severe artefact (Fig. 1) and score 5 to the most subtle (only diagnosed after measuring HU). We also reviewed the images when there were discrepancies in grading, which were resolved by consensus.

**Table 1:** Five-point scale for subjective rating of transient interruption of contrast (TIC)

Scale and score	Description of TIC
1	Severe, making CTPA nondiagnostic
2	Important, limiting the study although a PE diagnosis can eventually be made
3	Evident, with no significant reduction in the quality of the study
4	Visible, with slight visual differences but no effect on diagnostic accuracy
5	Not visually apparent, defined only by HU measurement differences greater than 10 HU

*CTPA* Coronary tomography pulmonary angiogram, *PE* pulmonary embolism

### Statistical analysis

Statistical analysis was performed with commercially available software (SPSS 15 for Windows; SPSS, Chicago, IL). Statistically significant differences were considered using a *P* value of <0.05. Normality of data distribution was assessed with Kolmogorov-Smirnov test.

Characteristics of the three patient groups—age, weight, height, and body mass index (BMI)—were compared by using the Student’s *t*-test. For sex, χ^2^ test was used. The χ^2^ test was also used to test the significance of differences regarding the presence of TIC and its grades among the groups. The Mann-Whitney *U*-test was applied to assess differences in age, weight, height, and BMI in patients with or without TIC.

## Results

### Patient characteristics

Comparison of the three patient groups did not reveal any significant differences in patient sex, age, weight, height, and BMI, except for a significantly older age in group C corresponding to noncooperative patients compared with A and B (*P* < 0.001).

Distribution in groups and demographic and anthropometric characteristics are summarised in Table 2.

**Table 2:** Patient characteristics

Parameter	Group A	Group B	Group C	*P* value
Male-to-female ratio	46:46	53:41	20:25	0.41
Age (years)	60 ± 18.9 (19–91)	65.7 ± 16.7 (18–92)	75.3 ± 11.8 (30–93)	<0.001
Weight (kg)	74.9 ±18	75.4 ± 15	72.1 ± 15.1	0.52
Height (cm)	165.4 ± 9.5	165.3 ± 8.6	163.1 ± 6.9	0.32
BMI (kg/m^2^)	27.4 ± 5.9	27.6 ± 5.3	26.9 ± 5	0.79

Data are presented as numbers or means ± standard deviations Values *in parentheses* are ranges

### Pulmonary attenuation

Means and standard deviations for main, left and right pulmonary artery densities for the three groups are shown in Table 3. Differences among the three groups were not significant.

**Table 3:** Pulmonary artery attenuation

Artery	Group A	Group B	Group C	*P* value
Main	345.7 ± 107.1	356.7 ± 128.1	393.8 ± 151.4	0.10
Right	334.8 ± 103.3	345.3 ± 123.5	364.3 ± 154.5	0.42
Left	320.3 ± 96.6	339.3 ± 129.8	368.6 ± 159.9	0.10

Data are means ± standard deviations in HU

### Incidence of TIC

**Fig. 2:**
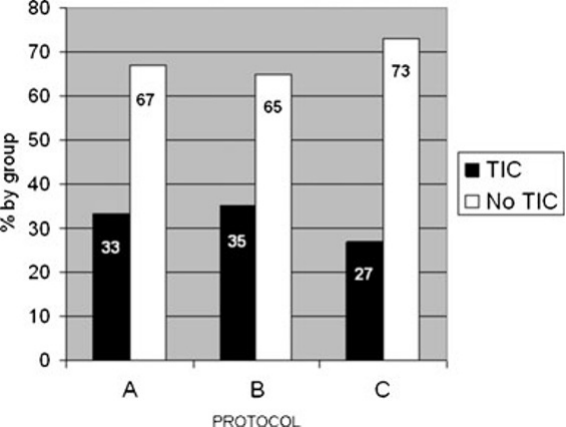
Percentage of CT scans showing transient interruption of contrast (TIC) by groups

Global incidence of any grade of TIC was 32.5% (75 of 231 patients). Distribution of TIC did not differ significantly among groups (*P* = 0.61): it was found in 30 (33%) of 92 patients in group A, 33 (35%) of 94 patients in group B, and 12 (27%) of 45 patients in group C (Fig. 2).

### Grade of TIC

**Fig. 3:**
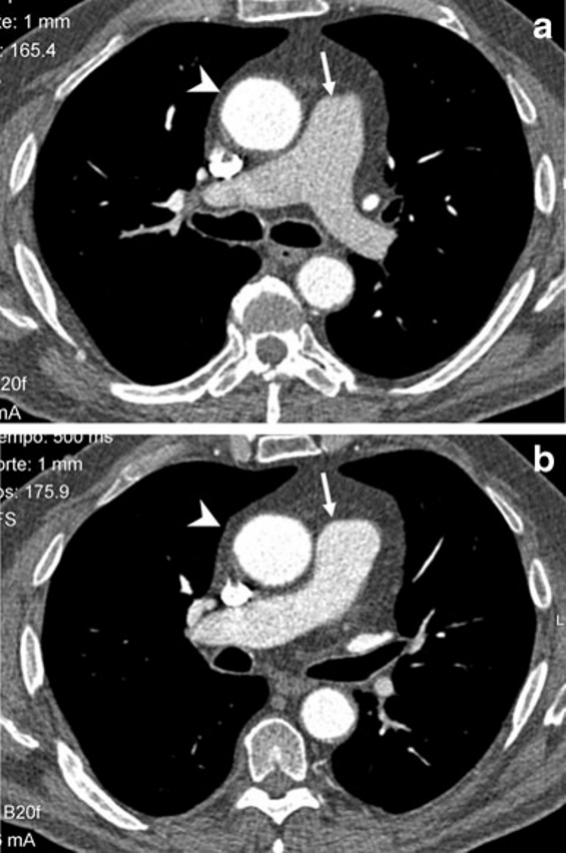
Transverse CT pulmonary angiogram at the level of the pulmonary trunk bifurcation (**a**) and 1 cm below (**b**) in a 59-year-old man. Pulmonary artery attenuation (*arrows*) is around 220 HU in **a** and shows higher density in **b** (around 300 HU), but the pulmonary artery is less enhanced than the aorta (*arrowhead*), pulmonary veins, and superior vena cava. Patient was considered as presenting a grade 3 transient interruption of contrast (TIC) by both observers

Most patients with TIC exhibited grade 4 (36 patients) followed by grade 3 (Fig. 3; 21 patients) and grade 5 (11 patients). Severe TIC (grades 1 or 2) was present only in five patients.

**Fig. 4:**
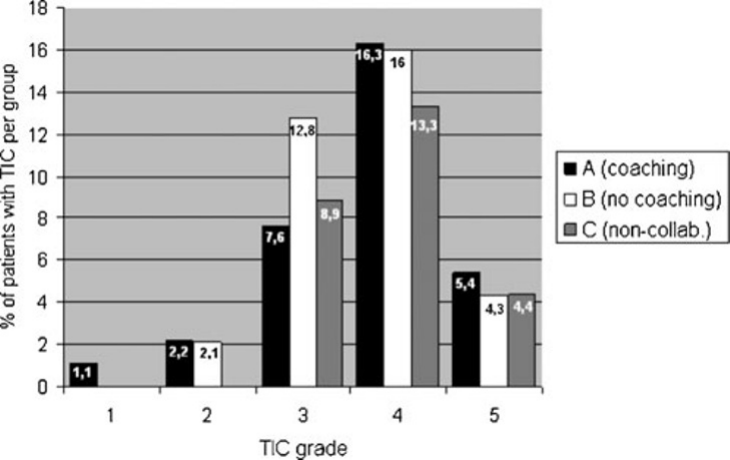
Percentage of patients with transient interruption of contrast (TIC) in each group, distributed by grades

There were no statistically significant differences in the distribution of these grades among the groups (*P* = 0.92) (Fig. 4 and Table 4).

**Table 4:** Number of patients with transient interruption of contrast (TIC) in each group, distributed by grades

Group	TIC grade				
	Grade 1	Grade 2	Grade 3	Grade 4	Grade 5
Group A: coaching (*n* = 92)	1	2	7	15	5
Group B: no coaching (*n* = 94)	–	2	12	15	4
Group C: noncooperative (*n* = 45)	–	-	4	6	2

### Incidence of significant TIC

TIC grades 1, 2, and 3 were considered together as evident TIC and potentially limiting the diagnosis of PE. Global incidence of this “significant” TIC was 12% (28 of 231 patients) and distribution by groups was as follows: 10 (11%) of 92 patients in group A, 14 (15%) of 94 patients in group B, and 4 (9%) of 45 patients in group C. When compared with the remaining patients with no TIC or TIC grades 4 or 5, differences were also nonsignificant (*P* = 0.53).

### Effect of inspiration on TIC

For further analysis, patients were divided into those who performed an inspiration before scan (irrespective of respiratory coaching or not) and those who did not. So, groups A and B were joined and compared with group C. Incidence of any grade of TIC was compared and a higher frequency of the phenomenon was found in the patients who made inspiratory effort, although the difference was not statistically significant: 63 (34%) of 186 vs. 12 (27%) of 45 (*P* = 0.35). The same occurred when only significant TIC was considered: 24 (13%) of 186 vs. 4 (9%) of 45 (*P* = 0.46).

## Discussion

CTPA has become the initial diagnostic test of choice to identify pulmonary embolism in most institutions, due to its availability, noninvasiveness, and rapid examination time. Despite the evolving technology of multidetector scanners that has improved its accuracy, achieving high sensitivity and specificity values, examinations are still being performed with poor image quality because of artefacts that may render a study nondiagnostic for the exclusion of a pulmonary embolus. The most common causes are motion artefacts, followed by poor enhancement [4, 10, 11]. Pulmonary artery attenuation can also be affected by a phenomenon known as TIC, which is a physiological artefact that is present when a segment of the pulmonary arteries shows decreased opacification, while other portions of the pulmonary arteries and/or the aorta show higher attenuation. Although uncommon, TIC is still a cause of nondiagnostic CTPA, with 2% of scans receiving the most severe score (1 and 2) in our study. Recently, TIC has been reported as a more frequent cause of nondiagnostic CTPA in pregnant women [12]. The reported incidence of TIC differs among authors. Gosselin et al. reported this artifact in 37% of their study group [5], whereas in Wittram and Yoo’s study population TIC was seen in 3% of cases [6]. Jones reported an incidence of 4.2% [11]. Finally, Mortimer et al. [7] reported incidences of 14 and 29% in inspiratory and expiratory scans, respectively.

The major difference between these publications, and a possible explanation for this variable incidence, is that the criteria used for diagnosis of TIC vary among studies. In Wittram’s study, the presence of an artefact was identified by visual inspection, and in cases with an artefact, the attenuation values were measured. On the other hand, in Gosselin’s and Mortimer’s studies, as in ours, the diagnosis was made by taking into account differences in HU values, which were measured in all patients. Owing to these methods, subtle artefacts could not be detected in Wittram’s study because they were not visually apparent, whereas in Gosselin’s and Mortimer’s populations, as in our study group, all artefacts that occurred were registered (including those cases only detectable by measuring attenuation values), resulting in a greater incidence of TIC. Moreover, by grading TIC in our study, we showed that an important percentage of cases had a slight artefact (grades 5 and 4): 63% of cases with TIC (47 of 75) and 20% of all cases (47 of 231 patients), whereas the incidence of visually apparent TIC (grades 3, 2, or 1) was 12% (28 of 231 patients), a figure more similar to that found in Wittram’s study. On the other hand, the global incidence of any grade of TIC in our group was 32.5%, which is closer to the percentage observed by Gosselin.

Both Wittram and Gosselin postulated that TIC can be related to a variable inflow of unopacified blood from the IVC into the right heart, as a normal response to negative intrathoracic pressure resulting from a deep inspiration made before the scan starts [5, 6]. Mortimer et al. [7] added a possible mechanism consisting of a reduction in flow of contrast medium from the superior vena cava (SVC). Although not exactly a Valsalva maneuver, sustained inspiratory effort and breath-hold can have similar complex hemodynamic effects that can lead to a substantial temporal reduction in contrast medium concentration at some level of the pulmonary arterial system.

Thus, the presumed solution to avoid the occurrence of TIC is to minimize either the volume of unopacified blood entering the right atrium from the IVC or the reduction of flow of opacified blood coming from the SVC. There is no agreement on the role of respiratory maneuvers to avoid these hemodynamic effects. While Gosselin et al. suggest the practice of hyperventilating the patient prior to the examination to reduce the mixing artefact [5], Wittram and Yoo propose that this prescanning hyperventilation may be actually a potential cause [6] and could explain the different incidence of TIC between their study population and Gosselin’s.

Another study performed by Chen et al. investigated if CTPA in the expiratory phase could reduce the effect of TIC [13]. They rescanned patients with indeterminate inspiratory CTPA due to this artefact in an expiratory phase, as expiration decreases negative intrathoracic pressure resulting in decreased systemic venous return to the right heart, with a relatively smaller contribution of unopacified blood from IVC. They concluded that expiratory imaging for these nondiagnostic CTPA improved pulmonary arterial enhancement, but they did not demonstrate it to be superior to inspiratory phase scanning for a general population. In a recent study [7], incidence of TIC was significantly reduced when expiratory phase scanning was compared with inspiratory phase scanning, and the severity of the artefact was judged as mild in all expiratory scans. No studies have been developed to compare whether performing different respiratory protocols with breath-hold in an inspiratory phase has an impact on the incidence of TIC.

What we call in our study “respiratory coaching” was previously performed so that the patient could sustain the inspiration during the required 20–30 s of scanning when a single-slice CT was used. With the advent of MDCT, there is no need for patient’s compliance due to shorter scan times. However, the possible positive effect on the incidence of TIC has not been previously evaluated.

Mean attenuation in pulmonary arteries did not differ significantly among the three protocols performed, however the mean attenuation was 40–50 HU higher for group C compared with A and B. Since group C did not perform a respiratory effort, it could be suggested that lack of deep inspiration and its Valsalva-like hemodynamic consequences are responsible for this greater attenuation.

We have failed in this study to show any positive or negative effect of any of the respiratory protocols evaluated. Even patients in group C, to whom no respiratory orders or just a shallow breathing order were given, showed several grades of TIC. The only positive effect in this group was that no patient exhibited the most severe grades of TIC, supporting again the role of deep inspiration just before the scan as a cause of it. Another potential condition described in the literature that can influence the interruption of the contrast bolus column is the presence of a patent foramen ovale (PFO) [9]. About 25–30% of people have a PFO without hemodynamic significance at rest. While performing a deep inspiration, an increased right atrial pressure can develop a short-term right-to-left shunt, so opacified blood will arrive directly to the left heart, bypassing pulmonary circulation. Though the incidence of PFO was not known in all our study patients, dedicated review showed no indirect signs of it by CT imaging in the most severe cases of TIC. Moreover, echocardiography failed to show a PFO in one patient with grade 2 and in the only patient with grade 1 TIC.

There are some limitations to our study. First, a typical flow rate of 3 mL/s was used during the study, and although we have not found a relationship between TIC incidence and flow rate, it can be argued that with higher rates the effect of TIC could be diminished. Second, the grade of compliance of an individual patient and the strength of the inspiratory efforts can be greatly affected by a number of factors. Although efforts were made to properly explain the respiratory protocols to all the patients and to ensure the fulfillment of these instructions, it is practically impossible to guarantee that all of them have breathed and performed the respiratory protocols in the same manner in each group. Third, the low incidence of severe TIC phenomenon is a major limitation to prove differences between groups, if they really exist. This fact must be taken into account when interpreting our results. In conclusion, performing respiratory coaching before inspiration for CT pulmonary angiography had no effect on the incidence of transient interruption of contrast phenomenon in this prospective randomised study.

## Abbreviations

CTPA: CT pulmonary angiogram
IVC: Inferior vena cava
PE: Pulmonary embolism
PFO: Patent foramen ovale
SVC: Superior vena cava
TIC: Transient interruption of contrast
